# Functional impacts of exoskeleton-based rehabilitation in chronic stroke: multi-joint versus single-joint robotic training

**DOI:** 10.1186/1743-0003-10-113

**Published:** 2013-12-19

**Authors:** Giuliana Grimaldi, Mario Manto

**Affiliations:** 1 Unité d’Etude du Mouvement, ULB-Erasme, 808 Route de Lennik, 1070 Bruxelles, Belgium; 2 FNRS, Bruxelles, Belgium

**Keywords:** Stroke, Rehabilitation, Exoskeleton, Robotics, Functional

## Abstract

Stroke is a major cause of disability in the world. The activities of upper limb segments are often compromised following a stroke, impairing most daily tasks. Robotic training is now considered amongst the rehabilitation methods applied to promote functional recovery. However, the implementation of robotic devices remains a major challenge for the bioengineering and clinical community. Latest exoskeletons with multiple degrees of freedom (DOF) may become particularly attractive, because of their low apparent inertia, the multiple actuators generating large torques, and the fact that patients can move the arm in the normal wide workspace. A recent study published in *JNER* by Milot and colleagues underlines that training with a 6-DOF exoskeleton impacts positively on motor function in patients being in stable phase of recovery after a stroke. Also, multi-joint robotic training was not found to be superior to single-joint robotic training. Although it is often considered that rehabilitation should start from simple movements to complex functional movements as the recovery evolves, this study challenges this widespread notion whose scientific basis has remained uncertain.

## 

The burden of stroke in the world is huge [1]. Stroke is the second leading cause of death in patients older than 60 years [2], and stroke survivors often suffer from long-term disabling deficits impacting on their quality of life. In a worldwide perspective, the impact in terms of healthcare costs is enormous. It is estimated that the direct costs of stroke range between 2 and 4% of the health expenses [3]. It is not surprising that numerous clinical and research centers in the world are trying to promote recovery by applying efficient methods of rehabilitation. Currently, most rehabilitation centers provide training and rehabilitation programs based on repetitive tasks [4], often with a functional feature [5]. Although there is a great hope that robotic devices will replace and/or assist efficiently physiotherapists, the implementation of rehabilitation based on robotic devices remains a major challenge for the bioengineering and clinical community [6,7]. This is due in particular to the limited degrees of freedom (DOF) of the devices currently available, their lack of ergonomy, and the fact that these techniques are often highly sophisticated. For the upper limb, functional tasks require multi-joint movements involving a high number of muscles, from the neck to the hand [8]. So far, the use of robotic devices has not clearly demonstrated that it impacts positively on the functional status of neurological patients, as compared to a conventional rehabilitation program handled by well trained and experienced physiotherapists [9]. However, with the advent of carefully designed exoskeletal devices, there is a resurging hope that the movements dictated by the robot will -nearly perfectly- mimick the natural movements which were performed by patients before the occurrence of the lesion(s) of the brain. The high number of repetitions allowed by robots is a concept attracting scientists for more than 2 decades, including for exoskeletons. Still, we have currently very few informations regarding the impact of these devices in terms of clinical recovery and functional outcomes. Brain imaging studies are encouraging. For instance, functional MRI studies have shown that robot-based therapy induces an increased sensorimotor cortex activation, with a task-specific reorganization of motor maps [10].

In a recent study published in *JNER*, Milot et al. report on a well designed study with a 6-DOF pneumatically-powered exoskeleton (BONES: Biomimetic Orthosis for the Neurorehabilitation of the Elbow and Shoulder), which can train the whole upper limb [11]. Interestingly, (1) the shoulder actuators are mechanically grounded to allow a low apparent inertia, and (2) multiple actuators acting in parallel allow to generate large torques. This exoskeleton has another advantage: patients can move the arm in the normal wide workspace. An assistance-as-needed algorithm was used by the authors. A key-issue addressed is whether exoskeletons do improve behavioral outcomes after a stroke. The authors applied randomly two different types of robotic training (multi-joint robotic training: MJRT, versus single-joint robotic training: SJRT) in chronic stroke survivors with unilateral lesions. Patients were in a stable phase of recovery after the stroke, so that the possible functional gains were attributed to the robotic training itself. A cross-over design was applied and patients were assessed by a blinded therapist. Sessions of one hour were repeated three times per week during one month. Functional tests used to assess the effects or MJRT/SJRT included the box and block test (BBT), the Fugl-Meyer Arm Motor Scale (FMA), the Wolf Motor Function Test WMFT, the Motor Activity Log (MAL). Quantitative measurements of strength and velocity of reaching were also assessed. These functional tests were performed at baseline, after each training period and after 3 months, with a total of 4 evaluations. Robotic training was associated with significant functional improvements, which remained after 3 months. Manual dexterity improved. However, no significant difference was found between MJRT and SJRT. In particular, MJRT was not superior to SJRT in terms of improvement of the BBT score. In a post-therapy survey, the patients replied that they enjoyed training with the robot. Patients had the feeling that robotic training impacted positively on the quality of movement. The rehabilitation community now agrees that the issue of patients’ satisfaction cannot be neglected, especially in the field of robotics.

This study highlights that (1) training with an exoskeleton impacts positively on motor function in chronic stroke, and (2) MJRT is not superior to SJRT. It is often considered that rehabilitation should start from simple movements to complex functional movements as the recovery evolves. The current study challenges this disseminated concept whose scientific grounds have remained unclear. This article underlines the importance of evidence-based stroke care. Future rehabilitation programs based on SJRT are likely to emerge in the coming years, using robots administering SJRT.

Future critical questions remain unsolved and should be addressed. Should we perform an exoskeleton-based RT *immediately* after the stroke to provide intense sensory feedback to the brain? Would this be associated with a faster functional recovery of multi-joint tasks? Should RT be primarily task-oriented? Is well designed RT-based rehabilitation superior to conventional therapy, or should RT become an adunct therapy administered to highly selected neurological patients? Should we move towards multimodal approaches to restore upper limb functions [12]? Which patients are likely to respond to RT and how physiotherapists can predict the percentage of functional response? The authors are now assessing the baseline variables predictive of positive functional gains [11]. Indeed, the identification of predictive factors would be a subsequent step towards a better RT-based rehabilitation care in stroke. Also, can we extend the present results to other diseases that affect the brain, such as traumatic brain injury (TBI) or other forms of acquired brain damage? This would expand dramatically the potential of exoskeleton-based RT. The recent developments of robot-mediated neurorehabilitation in rodent models of stroke will also increase our understanding of the mechanisms underlying clinical improvements in patients affected by this devastating disorder [13]. Restoration of upper limb function is more than ever a topic of research.

## Competing interests

The authors declare that they have no competing interests.

## Authors’ contributions

GG and MM prepared and drafted the manuscript. Both authors read and approved the final manuscript.
